# The Treatment of Typhoid Fever

**Published:** 1901-02-09

**Authors:** 


### HOSPITAL CLINICS AND MEDICAL PROGRESS.

### THE TREATMENT OF TYPHOID FEVER.

FROM many sides come signs of revolt against the tendency which has asserted itself so strongly during the past twenty years to reduce the treatment of typhoid fever to a mere routine, having for its central factor the use of a milk diet, and we cannot but think that Dr. Frederick J. Smith did good service in speaking as he did at the last meeting of the Medical Society, and in urging that the dieting of a patient must be arranged in accordance with the indications given by the appetite and the appearances of the stools. Not that we raise a word against milk in typhoid fever—for good milk is an admirable food. It is the routine that is the evil, and this applies to other things besides milk. When men tell us that we must not argue from individual cases, and assure us that if we could treat, say, 1,000 cases with milk, or with baths, or with what not, we should have better results than by dodging about after individual peculiarities, we feel sure they have got hold of the wrong end of the stick. Cases are not so similar as all that, and we think that Dr. Frederick Smith's address to the Medical Society is worthy of very serious consideration. His text is the study of the individual: — "The patient, or his body, is before us as an individual case with its own secrets and interests, and not as a unit in a row of figures with some subtle paper relationship to percentages." In the treatment of such cases there are two points of overwhelming importance. They are (1) the condition of the stools, and (2) the appetite of the patient. It may be taken as a golden rule, from which no exception should be made, that one stool at least must be carefully examined in every twenty-four hours during the active course of the disease. Even in private houses the stool should be carefully stirred about with something (that can be burnt), so that its composition may be investigated. In stools so examined there may be found (1) obviously undigested milk or other food; (2) blood; (3) sloughs; and (4) mere fæculent débris.

1. The presence of undigested milk or other food may be taken as conclusive proof that more is being given than can be digested, and is an absolute indication that the quantity must be considerably reduced. Dr. Frederick Smith's plan is to stop all food absolutely for 24 hours, and then begin with very small quantities indeed, and if it be milk that curds to prohibit milk in its natural raw state altogether.

2. If blood be visible to the naked eye, and yet be present only in small quantities, it must be taken as indicating considerable severity of intestinal lesion, and points to caution; but if it be present in large quantity opium must be given freely—say 10 minims every two hours for eight or ten doses, or until the patient is drowsy—and all food must be stopped absolutely for 24 or 48 hours, so as to give as complete rest as possible both to the mind and the intestine.

3. The appearance of sloughs about the third or fourth week is natural in a case of considerable severity, and their total amount suggests the extent of the existing ulceration. They make one anxious about hæmorrhage, and suggest starvation or caution about feeding for a day or two.

4. Fæculent débris forms the desirable constituent, and the more this is present without any of the other elements named above, the stronger is the indication to persist in the line of treatment which has been adopted.

The next point is that the appetite of the patient is to be taken as a guide to the feeding. Appetite means the readiness of the stomach for food, and, in the absence of dyspepsia, the capability of the stomach for dealing with appropriate articles put into it. Provided then that vomiting, hæmorrhage, or distension of the abdomen are absent, the appetite of the patient may within very wide limits be taken as the sole arbiter of his diet. The temperature may be entirely ignored in this matter, for if the wish for food exists the stomach is clean enough to deal with the articles enumerated below, so that what gets into the intestines will do no mechanical damage to their walls. On the other hand, when appetite is in complete abeyance food should not be given. The only food that can be dealt with then, is the one food generally most craved for by the patient—plain cold water. If we force food in it only travels slowly on, decomposing as it goes, and causing discomfort if not actual danger. "The idea of feeding the patient in the so-called typhoid stage—dry brown tongue, semi-delirium, &c.—on anything but water is to me," says Dr. Smith, "repugnant in the extreme." This sort of water diet may be carried on if necessary for four or five days. We are far too much afraid of starving our patients. But neither must starving be done by routine. The diet and the circumstances of each individual case must be carefully adjusted to each other.

Among what forms of diet, then, may we take our choice in managing a case of typhoid fever? Milk must be placed first, and as a basis for diet it must remain one's sheet-anchor. But it is never to be given in any bulk in its natural raw state. If so given it forms a tough ball of curd in the stomach very likely to set up vomiting. It should be given in such a form that this balling and curding is prevented; for example, as liquid custard, or as baked custard pudding broken up with a fork, or as junket, or as the basis of cocoa, coffee, tea, or bread and milk, or with rusks soaked in it and beaten up with a little baked fruit pulp, or as milk puddings with ground rice, sago, or tapioca. These foods should never be peptonised. Eggs, lightly boiled or poached, may be allowed at any stage, or at anyrate the yolk spread on bread or a rusk. Jelly and soups are often grateful to the palate. Home made, cold drawn beef-tea may be given, or beef juice obtained by quickly cooking a steak over a very hot fire, splitting it open, and scraping with a sharpish spoon, so as to obtain tiny fragments of muscle with the juice. Meat or fish must be pounded or passed through the sausage machine. Bread (very stale), rusks, or biscuits may be nibbled at, or may be taken soaked in milk. Beer or stout given as a stimulating food need never be denied to a patient who asks for it, but other forms of alcohol given purely for their stimulating effect come under the heading of drugs, and are rarely necessary. In the typhoid state a patient who likes bottled beer may have it in place of water as the sole food to be taken. The only articles to be avoided are those which leave small hard particles after leaving the stomach, such as raw fruit with their pips and skins; the juice there is no objection to. When, although the fever is developed, the patient is conscious, five meals in the twenty-four hours should suffice: every four hours during the day, and one meal in the night if the patient is awake—say between midnight and 4 A.M. No meal should exceed a cupful in quantity, and one of them may consist of a couple of ounces of custard, and another of two tablespoonfuls of crushed meat or fish. Between the meals nothing should be given but cold water, or hot water if the patent prefer it.

Vomiting in typhoid fever is a very exhausting complication, and usually arises from routine feeding against the feelings of the patient. On the first indication of nausea after food resort should at once be had to plain water diet for twelve hours, and longer if necessary. Bismuth, soda, and hydrocyanic acid may be given if it persist. Tympanites is of the most ominous significance. If it is due to perforation and peritonitis surgery affords the sole chance, and a very poor one. But occurring by itself there is good ground for believing that it is the result of improper feeding. The slightest rigidity of the abdominal muscles or the slightest convexity of the abdomen beyond its natural condition should make us immediately stop all food except water, and two drachms of sulphate of soda in warm water should be given every two hours till the bowels are acting freely. An ice-bag may be placed on the abdomen, and chlorine water or salol may be given as antiseptics. Constipation should never be allowed to go on, and sulphate of soda is the remedy, aided, if necessary, by an enema to start it. Excessive diarrhœa is commonly the result of improper feeding. Whenever it occurs the stools should be most carefully inspected. They may show undigested food, in which case the treatment is obvious. On the other hand, they may only show much sloughy matter from the intestines, with large quantities of mucus. The cause of this is often extensive ulceration of the colon. Excessive diarrhœa not due to improper feeding indicates then a very serious condition, and is thus a very fatal complication. In regard to the treatment of hæmorrhage, Dr. Smith says that in his opinion if starvation and opium are found to be useless, nothing else is likely to be of the slightest use. Perhaps we may venture to add that where hæmorrhage occurs along with tympanites the indication for emptying the bowels, either as Dr. Smith describes by sulphate of soda or even by a dose of castor oil, is all the stronger. Hæmorrhage may occur from the ulcerative process eating into a vessel, and in the presence of such an event we are very helpless. But any ulcer, even while healing, may bleed in consequence of being stretched by tympanites, and for this we can do a good deal.

As to drugs, calomel at the beginning and a mixture containing chlorine water, or carbolic acid may be given, or 10 grains of salol three times a day. Alcohol may be considered as unnecessary for the disease; but to those patients who are in the habit of taking it, it may be given, and generally in the form to which they are accustomed.

